# Expression of metallothionein and Nrf2 pathway genes in lung cancer and cancer-surrounding tissues

**DOI:** 10.1186/1477-7819-11-199

**Published:** 2013-08-16

**Authors:** Gui-You Liang, Sheng-Xun Lu, Gang Xu, Xing-Da Liu, Jian Li, Deng-Shen Zhang

**Affiliations:** 1 Affliated Hospital of Zunyi Medical College, Department of Thoracic and Cardiovascular Surgery, Zunyi, Guizhou 563003, China

**Keywords:** Lung cancers, Cancer-surrounding tissue, Nrf2, Metallothionein

## Abstract

**Background:**

Nuclear factor (erythroid-derived 2)-like (Nrf)2 and metallothionein have been implicated in carcinogenesis. This study investigated the expression of Nrf2 and of Nrf2-targeted genes (*NQO1* and *GCLC*) and the genes for the metallothionein (MT) isoforms (MT-1A and MT-2A) in human lung cancer and cancer-surrounding tissues.

**Methods:**

Surgically removed lung cancer samples (n = 80) and cancer-surrounding tissues (n = 38) were collected from Zunyi Medical College Hospital, China. Total RNA was extracted, purified, and used for real-time reverse transcription-PCR analysis of interested genes.

**Results:**

Expression of the Nrf2-targed genes *NQO1* and *GCLC* tended to be higher (30 to 60%) in lung cancers, but was not significantly different from that in peri-cancer tissues. By contrast, expression of the genes for M)-1A, MT-2A, and the metal transcription factor MTF-1 were three-fold to four-fold lower in lung cancers.

**Conclusion:**

In surgical samples of lung cancer, *MT* expression was generally downregulated, whereas *Nrf2* expression tended to be upregulated. These changes could play an integral role in lung carcinogenesis.

## Background

Nuclear factor (erythroid-derived 2)-like 2 (Nrf2) is a transcription factor belonging to the ‘cap “n” collar’ subfamily of the basic-leucine zipper (bZIP) family of transcription factors, which plays a significant role in adaptive responses to oxidative stress [1]. Activation of Nrf2 can have good, bad, and ugly effects in biology, especially during carcinogenesis [1,2]. However, little is known about the role of NRF2 expression in surgically removed lung cancers.

Metallothioneins (MTs) are a group of low-molecular weight, cycteine-rich, metal-binding proteins, which are encoded by a family of genes located at 16q13. This family of proteins consists of 10 functional isoforms in humans, with MT-1A and MT-2A being the predominant forms [3]. It has been shown that aberrant expression of MTs is related to tumor type and different stages of tumor development and progression [3,4].

Hypermethylation of human MT isoforms and reduced MT gene expression are frequently seen in hepatocellular carcinoma (HCC) [5-7]. Both increased [8] and suppressed [9] MT expression have been reported in lung cancer compared with normal lungs. However, little is known about the expression of MT in lung tumors and peri-tumor tissues.

MT is silenced via methylation status changes [5]. Methylation of MT-1A and MT-2A in malignant mesothelioma was shown to be associated with tumor grade histology and lymph-node involvement [10]. MT protein stained positively in lung adenocarcinoma, but was absent in small cell lung carcinoma [11], suggesting that MT expression in the lung is tumor type-specific.

To further explore the role of Nrf2 and MT expression in lung carcinogenesis, this study used surgically removed lung cancer samples and available cancer-surrounding tissues to examine expression of these antioxidant components. Downregulation of MT-1A and MT-2A was found in the surgical stage of lung cancers, whereas the NRF2-targeted gene *NQO1* tended to increase. These gene expression changes could play an integrated role in lung carcinogenesis.

## Methods

### Study population

Lung cancer samples were obtained from specimens removed surgically during the period March 2008 to May 2009 at Zunyi Medical College Hospital (Guizhou, China). In total, 80 lung cancer specimens, both benign and malignant tumors, were collected, together with 38 available cancer-surrounding tissues.

### Ethics

All the human studies were approved by the Institutional Human Subject Study Committee of Zunyi Medical College Hospital. All patients were informed and signed a consent to allow to use the surgical specimens for scientific research.

### RNA isolation

Total RNA was extracted (Trizol reagent; Huashun Bioengineering Co, Shanghai, China) in accordance with the manufacturer’s instructions. RNA quality and quantity was determined spectrometrically, with a 260/280 nm ratio of greater than 1.8.

### Real-time reverse transcription-PCR analysis of Nrf2 and MT

Total RNA was then used for real-time reverse-transcription (RT)-PCR and specific cDNAs were amplified (SYBR® PrimeScriptTM RT-PCR Kit; TaKaRa, Dalian, China). The Nrf2 and MT isoform primers were designed with Primer3 software (version 4.0), and are shown in Table 1. Real-time PCR was performed using a real-time PCR System (IQ5; Bio-Rad Laboratories, Inc., Hercules, CA, USA) in a 96-well optical plate format. The relative differences in expression between groups were expressed using cycle time (Ct) values. The Ct values of the interested genes were first normalized to β-actin in the same sample, and then the relative differences between the control and treatment groups were calculated and expressed as relative increases, setting controls as 100%.

**Table 1:** Primer sequences for real-time RT-PCR

**Gene**	**GenBank nyu**	**Forward**	**Reverse**
GADPH	NM_002046	ACAGTCAGCCGCATCTTCTT	ACGACCAAATCCGTTGACTC
GCLC	NM_001498	GTGGATGTGGACACCAGATG	GCGATAAACTCCCTCATCCA
MT-1A	NM_005946	GCAAATGCAAAGAGTGCAAA	CAGCTGCACTTCTCTGATGC
MT-1E	NM_175617	GGGCTTTCTTTGCCCTCATT	CTGTCCTGCCCCATCTGAAT
MT-1G	NM_005950	CCTGTGCCGCTGGTGTCT	TGCAGCCTTGGGCACACT
MT-2A	NM_005953	GTGTGCCCAAGGCTGCAT	TTGTGGAAGTCGCGTTCTTTAC
MT3	NM_005954	AGTGCGAGGGATGCAAATG	GCCTTTGCACACACAGTCCTT
MT-4	U07807	TCCAGGCCTCATGTGATTCAC	CCCTCTTGGCTAGGCACAGT
MTF-1	NM_005955	GCGAGTGCACACGAAGGA	CTGATGTGCTTTCAGCCTGTACA
NQO1	NM_000903	GTTGCCTGAAAAATGGGAGA	AAAAACCACCAGTGCCAGTC
NRF2	NM_006164	CGGTATGCAACAGGACATTG	GTTTGGCTTCTGGACTTGGA

### Statistical analysis

Data are expressed as mean ± SEM. The SPSS statistical program (version11.5 for Windows; SPSS Inc., Chicago, IL, USA) was used for ANOVA, followed by Turkey’s multiple comparison tests. *P* < 0.05 was considered significant.

## Results

### NRF2 and NRF2 target genes

**Figure 1:**
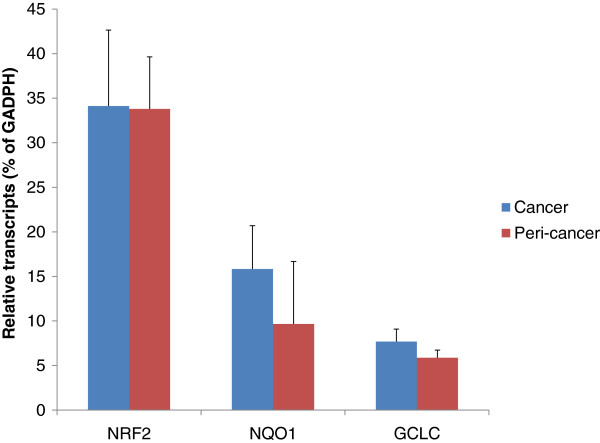
**Expression of the nuclear factor (erythroid-derived)-like (Nrf)2 and Nrf2-targeted gene *NQO1 *and *GCLC *in human lung cancer (n = 62) and cancer-surrounding tissues (n = 21).**

Expression of NRF2 was generally unchanged (34.12 ± 8.52 in lung cancer versus 33.80 ± 5.84 in peri-cancer tissues). Expression of the NRF2-target genes *NQO1* (15.84 ± 4.85 versus 9.67 ± 2.01) and *GCLC* (7.68 ± 1.41 versus 5.88 ± 0.85) tended to increase, but was not significant because of very large individual variations (Figure 1).

### MT-1A, MT-2A, and MTF1

**Figure 2:**
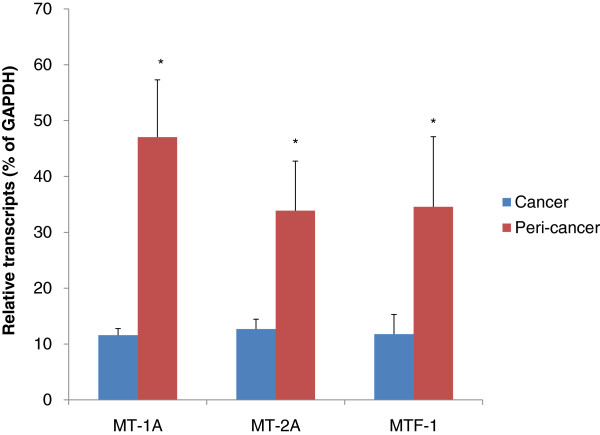
**Expression of metallothionein (MT)-1A, MT-2A, and metal transscription factor (MTF)1 in human lung cancers (n = 80) and cancer-surrounding tissues (n = 38).** *Significantly different from lung cancers, *P* < 0.05.

Expression of MT-1A and MT-2A in lung cancer and surrounding tissues are shown in Figure 2. MT-1A and MT-2A are the two most abundant MT isoforms in the lung. Expression of MT-1A mRNA was decreased four-fold in lung cancers (11.59 ± 1.16 in lung cancer versus 47.03 ± 10.26 in peri-cancer tissues. Expression of MT-2A followed a similar pattern, being approximately three-fold lower in lung cancers (12.68 ± 1.76 versus 33. 88 ± 8.87). Expression of MTF-1, a transcription factor for MT biosynthesis, was also lower in tumor compared with peri-cancer tissues (11.76 ± 3.52 versus 34.56 ± 12.56).

### Other MT isoforms

Expression of MT-3 and MT-4 was very low (0.35 and 0.41, respectively), and there was no difference in lung cancer compared with cancer-surrounding tissues (Table 2). Regarding MT isoforms, MT-1E and MT-1G were also downregulated in lung cancer tissues (Table 2), consistent with their methylation status and reduced expression in malignancies [12-14].

**Table 2:** **Expression of MT isoforms in lung cancers and cancer-surrounding tissues**^**a,b**^

	**Lung cancer tissue**	**Peri-cancer tissue**
MT-1A	11.59 ± 1.16^c^	47.03 ± 0.26
MT-1E	0.52 ± 0.23^c^	4.16 ± 0.96
MT-1G	0.66 ± 0.18^c^	1.71 ± 0.55
MT-2A	12.68 ± 1.76^c^	33.88 ± 8.87
MT-3	0.86 ± 0.27	1.52 ± 0.72
MT-4	0.06 ± 0.02	0.18 ± 0.07
MTF1	11.76 ± 3.52^c^	34.56 ± 12.56

Abbreviations: *MT* metallothionein; *MTF* Metal transcription factor.

^a^Total RNA was extracted from surgically removed lung cancers (n = 62 to 80) and cancer-surrounding tissues (n = 21 to 38), and the expression of MT isoforms was examined via real-time reverse transcription (RT)-PCR.

^b^Data are expressed as a percentage of the expression of the housekeeping gene glyceraldehyde 3-phosphate dehydrogenase.

^c^Significantly different from cancer-surrounding tissues p < 0.05.

## Discussion

In the present study, we used surgically removed lung cancer and cancer-surrounding tissues to examine the transcript levels of the two major antioxidant pathways, the Nrf2 pathway and MT molecules. The results clearly showed downregulation of MT isoforms in surgically removed lung tumors compared with the corresponding tumor-surrounding tissues. There was no difference in expression of Nrf2 between tumor and peri-cancer tissues, but the Nrf2 targeted genes *NQO1* and *GCLC* tended to be higher in lung cancer tissues.

The role of MT in lung cancers is dependent on the type and stage of tumor development [3]. In animal studies, MT stained negative in diethylnitrosamine-induced lung cancers [15,16], and deficiency of MT makes MT-null mice more susceptible to chemical-induced lung tumors [17].

All these experimental studies suggest that MT plays an important role in host defense against lung cancer development, and reduced MT expression is frequently associated with malignancies, such as HCC [3-5] and lung cancers [9]. Suppressed MT expression is related to epigenetic mechanisms such as MT gene methylation. Indeed, MT gene methylation ia evident in both human lung cancer [9] and HCC [3-5]. The methylation status of MT in lung cancer warrants further investigation. Large discrepancies in MT expression exist between different tumor types, and no distinct and reliable association exists between MT-1A and MT-2A expression in tumor tissues.

The roles of MT expression in tumor prognosis and therapy resistance are a matter debate. For example, in one study, MT positivity was obvious in 32 of 43 (74%) cases of squamous cell lung carcinoma, and in 12 of 35 (34%) cases of adenocarcinoma, whereas it was negative in all 11 cases of small cell lung carcinoma examined [11]. The different patterns of MT expression may relate to the antioxidant function of the protein in protecting against toxic stimuli [4]. A very large individual variation in MT expression also exists. In the present study, the difference in MT isoform expression between individuals was over 100-fold, and polymorphism of MT may dispose individuals to lung cancer development and progression. These possible links warrant further investigation.

Nrf2 is a transcription factor that positively regulates the basal and inducible expression of a large battery of cytoprotective genes. These gene products include proteins that catalyze oxidant reduction reactions (NQO1), glutathione synthesis (GCLC), and conjugation reactions (glutathione-S-transferase), and the efflux of potentially toxic xenobiotics and xenobiotic conjugates [18]. Thus, expression of the Nrf2-dependent proteins is crucial for ameliorating or eliminating toxicants/carcinogens to maintain cellular redox homeostasis. In addition, Nrf2 and Nrf2-targeted gene overexpression could also be related to abnormal expression of Kelch-like ECH-associated protein 1 [19]. In general, NRF2 is the cellular mechanism of cell survival. However, the ‘dark’ side of Nrf2 is that the damaged cells could escape clearance, allowing them to proliferate to produce cancer [20]. Nrf2 and its downstream genes are overexpressed in many cancer cell lines and human cancer tissues, giving cancer cells an advantage for survival and growth [2,20]. Thus, Nrf2-targeted gene overexpression in lung cancers could be a mechanism of lung carcinogenesis [1,2,20].

## Conclusions

In the current study, we found downregulation of MT isoforms in human lung cancers, especially in malignant tumors compared compared with cancer-surrounding tissues. By contrst, the Nrf2 targeted genes *NQO1* and *GCLC* tended to increase. All these changes could play an intergral role in lung carcinogenesis.

## Abbreviations

GCLC: Glutathione synthesis; HCC: Hepatocellular carcinoma; MT: Metallothionein; MTF: Metal transcription factor; Nrf: Nuclear factor (erythroid-derived)-like; NQO1: NAD(P)H: quinone oxidreductase; RT: Reverse transcription.

## Competing interests

The authors declare that they have no competing interests.

## Authors’ contributions

G-YL was reponsible for study concept and design; S-XL for data acquisition; G-YL and S-XL for data analysis and interpretation; S-XL, GX, and X-DL for statistical analysis; and JL and D-SZ for manuscript preparation. All authors have read and approved the final manuscript.

## References

[B1] Müller T Hengstermann A Nrf2: friend and foe in preventing cigarette smoking-dependent lung disease Chem Res Toxicol 2012 11 1805 1824 10.1021/tx300145n 22686525

[B2] Pi J Freeman ML Yamamoto M Nrf2 in toxicology and pharmacology: the good, the bad and the ugly? Toxicol Appl Pharmacol 2010 11 1 3 10.1016/j.taap.2010.01.005 20079756 PMC2837784

[B3] Cherian MG Jayasurya A Bay BH Metallothioneins in human tumors and potential roles in carcinogenesis Mutat Res 2003 11 201 209 10.1016/j.mrfmmm.2003.07.013 14643421

[B4] Klaassen CD Liu J Choudhuri S Metallothionein: an intracellular protein to protect against cadmium toxicity Ann Rev Pharmacol Toxicol 1999 11 267 294 10.1146/annurev.pharmtox.39.1.267 10331085

[B5] Jacob ST Majumder S Ghoshal K Suppression of metallothionein-I/II expression and its probable molecular mechanisms Environ Health Perspect 2002 11 Suppl 5 827 830 12426140 10.1289/ehp.02110s5827 PMC1241254

[B6] Kanda M Nomoto S Okamura Y Nishikawa Y Sugimoto H Kanazumi N Takeda S Nakao A Detection of metallothionein 1G as a methylated tumor suppressor gene in human hepatocellular carcinoma using a novel method of double combination array analysis Int J Oncol 2009 11 477 483 19639168 10.3892/ijo_00000359

[B7] Tao X Zheng JM Xu AM Chen XF Zhang SH Downregulated expression of metallothionein and its clinicopathological significance in hepatocellular carcinoma Hepatol Res 2007 11 820 827 10.1111/j.1872-034X.2007.00113.x 17517078

[B8] Dziegiel P Jeleń M Muszczyńska B Maciejczyk A Szulc A Podhorska-Okołów M Cegielski M Zabel M Role of metallothionein expression in non-small cell lung carcinomas RoczAkad Med Bialymst 2004 11 Suppl 1 43 45 15638370

[B9] Zhong S Fields CR Su N Pan YX Robertson KD Pharmacologic inhibition of epigenetic modifications, coupled with gene expression profiling, reveals novel targets of aberrant DNA methylation and histone deacetylation in lung cancer Oncogene 2007 11 2621 2634 10.1038/sj.onc.1210041 17043644

[B10] Tsou JA Galler JS Wali A Ye W Siegmund KD Groshen S Laird PW Turla S Koss MN Pass HI Laird-Offringa IA DNA methylation profile of 28 potential marker loci in malignant mesothelioma Lung Cancer 2007 11 220 230 10.1016/j.lungcan.2007.06.015 17659810 PMC2752414

[B11] Theocharis S Karkantaris C Philipides T Agapitos E Gika A Margeli A Kittas C Koutselinis A Expression of metallothionein in lung carcinoma: correlation with histological type and grade Histopathology 2002 11 143 151 10.1046/j.1365-2559.2002.01325.x 11952858

[B12] Faller WJ Rafferty M Hegarty S Gremel G Ryan D Fraga MF Esteller M Dervan PA Gallagher WM Metallothionein 1E is methylated in malignant melanoma and increases sensitivity to cisplatin-induced apoptosis Melanoma Res 2010 11 392 400 20848733

[B13] Ferrario C Lavagni P Gariboldi M Miranda C Losa M Cleris L Formelli F Pilotti S Pierotti MA Greco A Metallothionein 1G acts as an oncosupressor in papillary thyroid carcinoma Lab Invest 2008 11 474 481 10.1038/labinvest.2008.17 18332874

[B14] Henrique R Jerónimo C Hoque MO Nomoto S Carvalho AL Costa VL Oliveira J Teixeira MR Lopes C Sidransky D MT1G hypermethylation is associated with higher tumor stage in prostate cancer Cancer Epidemiol Biomarkers Prev 2005 11 1274 1278 10.1158/1055-9965.EPI-04-0659 15894685

[B15] Waalkes MP Diwan BA Weghorst CM Ward JM Rice JM Cherian MG Goyer RA Further evidence of the tumor-suppressive effects of cadmium in the B6C3F1 mouse liver and lung: late stage vulnerability of tumors to cadmium and the role of metallothionein J Pharmacol Exp Ther 1993 11 1656 1663 8371163

[B16] Waalkes MP Diwan BA Rehm S Ward JM Moussa M Cherian MG Goyer RA Down-regulation of metallothionein expression in human and murine hepatocellular tumors: association with the tumor-necrotizing and antineoplastic effects of cadmium in mice J Pharmacol Exp Ther 1996 11 1026 1033 8627513

[B17] Majumder S Roy S Kaffenberger T Wang B Costinean S Frankel W Bratasz A Kuppusamy P Hai T Ghoshal K Jacob ST Loss of metallothionein predisposes mice to diethylnitrosamine-induced hepatocarcinogenesis by activating NF-kappaB target genes Cancer Res 2010 11 10265 10276 10.1158/0008-5472.CAN-10-2839 21159647 PMC3059562

[B18] Klaassen CD Reisman SA Nrf2 the rescue: effects of the antioxidative/electrophilic response on the liver Toxicol Appl Pharmacol 2010 11 57 65 10.1016/j.taap.2010.01.013 20122946 PMC2860427

[B19] MacLeod AK McMahon M Plummer SM Higgins LG Penning TM Igarashi K Hayes JD Characterization of the cancer chemopreventive NRF2-dependent gene battery in human keratinocytes: demonstration that the KEAP1-NRF2 pathway, and not the BACH1-NRF2 pathway, controls cytoprotection against electrophiles as well as redox-cycling compounds Carcinogenesis 2009 11 1571 1580 10.1093/carcin/bgp176 19608619 PMC3656619

[B20] Lau A Villeneuve NF Sun Z Wong PK Zhang DD Dual roles of Nrf2 in cancer Pharmacol Res 2008 11 262 270 10.1016/j.phrs.2008.09.003 18838122 PMC2652397

